# A Rare Adverse Effect of the COVID-19 Vaccine on Autoimmune Encephalitis

**DOI:** 10.3390/vaccines10071114

**Published:** 2022-07-13

**Authors:** Ying-Fong Huang, Tzu-Chuan Ho, Chin-Chuan Chang, Daniel Hueng-Yuan Shen, Hung-Pin Chan, Kuo-Pin Chuang, Yu-Chang Tyan, Ming-Hui Yang

**Affiliations:** 1 Department of Nuclear Medicine, Kaohsiung Medical University Hospital, Kaohsiung 807, Taiwan; huangyf@kmu.edu.tw (Y.-F.H.); chinuan@kmu.edu.tw (C.-C.C.); 2 Department of Medical Imaging and Radiological Sciences, Kaohsiung Medical University, Kaohsiung 807, Taiwan; r090340@kmu.edu.tw; 3 School of Medicine, Kaohsiung Medical University, Kaohsiung 807, Taiwan; 4 Neuroscience Research Center, Kaohsiung Medical University, Kaohsiung 807, Taiwan; 5 Department of Electrical Engineering, I-Shou University, Kaohsiung 840, Taiwan; 6 Department of Nuclear Medicine, Kaohsiung Veterans General Hospital, Kaohsiung 813, Taiwan; hyshen@vghks.gov.tw (D.H.-Y.S.); hpchan@vghks.gov.tw (H.-P.C.); 7 Graduate Institute of Animal Vaccine Technology, College of Veterinary Medicine, National Pingtung University of Science and Technology, Pingtung 912, Taiwan; kpchuang@g4e.npust.edu.tw; 8 Graduate Institute of Medicine, College of Medicine, Kaohsiung Medical University, Kaohsiung 807, Taiwan; 9 Department of Medical Research, Kaohsiung Medical University Hospital, Kaohsiung 807, Taiwan; 10 Center for Cancer Research, Kaohsiung Medical University, Kaohsiung 807, Taiwan; 11 Research Center for Environmental Medicine, Kaohsiung Medical University, Kaohsiung 807, Taiwan; 12 Department of Medical Education and Research, Kaohsiung Veterans General Hospital, Kaohsiung 813, Taiwan; 13 Center of General Education, Shu-Zen Junior College of Medicine and Management, Kaohsiung 821, Taiwan

**Keywords:** COVID-19 vaccine, adverse effect, short-term memory loss, autoimmune encephalitis, lymphocytic pleocytosis

## Abstract

Since countries commenced COVID-19 vaccination around the world, many vaccine-related adverse effects have been reported. Among them, short-term memory loss with autoimmune encephalitis (AE) was reported as a rare adverse effect. Since case numbers are limited, this brief report may draw the attention of the medical community to this uncommon adverse effect and serve as a reference for future vaccine improvement. However, given the high risk of adverse outcomes when infected with SARS-CoV-2 and the clearly favorable safety/tolerability profile of existing vaccines, vaccination is still recommended.

## 1. Introduction

Coronavirus disease 2019 (COVID-19) is a new public health crisis affecting populations worldwide. Since the outbreak of COVID-19 in late December 2019, more than 539.8 million cases and more than 6.3 million deaths have been reported worldwide [1]. The main treatment options for COVID-19 are antiviral drugs (to prevent viral replication) and immunomodulatory/anti-inflammatory therapy (to avoid tissue damage). Most treatments are for symptomatic relief, with none ideal [2,3]. Thus, prevention (vaccination) is valuable. Comprehensive immunization with vaccines is considered a key strategy against SARS-CoV-2 [4]. Immunization may bring an end to the COVID-19 pandemic through global herd immunity. As a result, vaccine development was initiated through various platforms in 2020. As of the end of June 2022, more than 66.4% of the world population have received at least one dose of a COVID-19 vaccine, 12.02 billion vaccine doses worldwide have been administered and 5.61 million are now administered each day. However, only 17.8% of people in low-income countries have received at least one dose [5].

Since large-scale global vaccination began, different adverse effects and complications have been reported. Although important post-immunization surveillance of vaccines is ongoing, SARS-CoV-2 vaccines are currently considered highly effective and safe, even with neurological adverse events following immunization (AEFI) [6]. These transient symptoms include cerebral venous sinus thrombosis, demyelinating episodes, lymphadenopathy, nausea, cognitive decline, and localized swelling, erythema, pain, fever/chills, fatigue, headache, dizziness, muscle weakness, and myalgia/arthralgia [7]. However, we noticed that a small number of vaccine recipients developed adverse manifestations of transient amnesia, memory loss or disturbance. A minority of patients suffered from encephalitis or stroke after vaccination. In addition, most clinical examinations, including brain MRI, especially of Ankle/Brachial Index (ABI), Doppler, Electroencephalography (EEG), brainstem auditory EPs (BAPE), Polysomnography (PSG) and Holter EKG, revealed normal results for patients who experienced amnesia symptoms after injection. Variable neurological complications, despite the unproven causality, have also been reported in patients after receiving the COVID-19 vaccine, such as functional neurological disorder (symptoms of functional neurological disorder include muscle weakness, fatigue, cognitive impairment, dizziness, and impaired gait), facial palsy, Guillain–Barré syndrome (GBS), seizures, strokes, transverse myelitis, chronic fatigue syndrome, autoimmune encephalitis (AE), acute disseminated encephalomyelitis (ADEM), and acute encephalopathy. This mechanism of the neurological adverse effects is still unknown, but this is a phenomenon that requires further attention [8,9,10,11,12,13,14,15,16,17,18].

This is a new issue related to the COVID-19 pandemic and, in this paper, we explore autoimmune enthesopathy on COVID-19 vaccination. Although cases are rare, we searched for published articles that mention and discuss the issue of AE. To determine the mechanism of AE caused by COVID-19 vaccination, further study is needed.

## 2. Experimental Method

A literature review was performed on studies up to 25 June 2022 by searching in the electronic databases PubMed and Web of Science. We used the following search strings: “COVID-19 vaccination” with “forgetfulness or memory impairment or anterograde amnesia or transient amnesia or memory lose”. Variations of these terms were also searched. We also used the “related articles” option on the PubMed Web search homepage as well as manually searching through references listed in retrieved articles. Articles were limited to those with titles and abstracts available in English published in peer-reviewed journals. After deduplication, the identified full-text articles were examined for original data, and we also retrieved and checked for related references for further studies. Articles with the following criteria were included: (1) full-text articles that can be obtained from electronic databases; (2) patients enrolled in the studies were all laboratory confirmed for COVID-19 vaccination; and (3) articles included case reports.

## 3. Result and Discussion

From 2019 to the present, the rapid spread of COVID-19 has resulted in a global pandemic for which a vaccine was quickly developed. As the safety of COVID-19 vaccines continues to be monitored, cases of autoimmune encephalitis following vaccination with short-term memory loss have been reported. Short-term memory loss is a clinical syndrome characterized by the sudden onset of anterograde amnesia with autoimmune disorders, which are characterized by inflammation. Patients may develop cognitive impairment, which is referred to as autoimmune dementia. Autoimmunity can be caused by the following processes in patients: (1) molecular mimicry between infectious antigens and self-antigens, (2) the acceleration of the autoimmune process by antigen-presenting cells and antigens induced by foreign antigens, and (3) increased cytokine production and induced autoreactive T-cell expansion by B lymphocytes or bystander activation [19]. The immune system may attack healthy cells and tissues in the brain or spinal cord. Symptoms often vary from patient to patient. They can include a sudden decline in work or school performance, loss of the ability to speak, abnormal body movements or seizures, vision loss, weakness of the arms or legs, and sleep problems.

The long-term sequelae of SARS-CoV-2 infection are being increasingly recognized. These include cardiovascular disease, chronic lung disease, and proinflammatory-related neurological dysfunction that may lead to neurocognitive and psychological impairments. A major component of cognitive impairment is operationally classified as “brain fog”, which includes forgetfulness, difficulty concentrating, depression, fatigue and confusion. Short-term memory loss caused by autoimmunity after vaccination is somewhat like “vaccine brain fog” but the symptoms subside faster and the impact is smaller.

**Figure 1:**
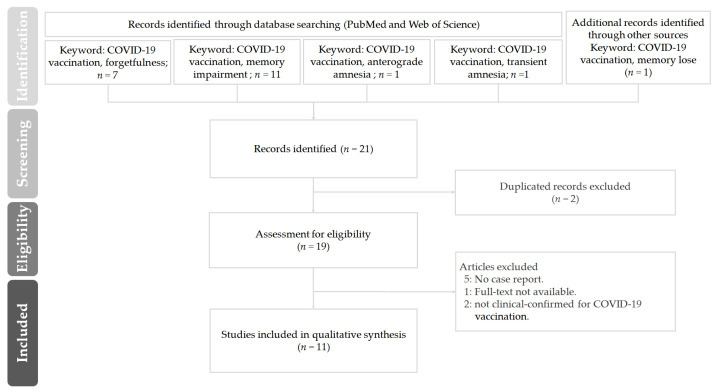
The PRISMA flow diagram of the literature review in this study on “forgetfulness or memory impairment or anterograde amnesia or transient amnesia or memory lose” on COVID-19 vaccination.

In this study, the literature search commenced in 2021, and the last day of the search was 24 June 2022. Figure 1 shows the PRISMA flow diagram of the literature review to identify clinical studies regarding “forgetfulness or memory impairment or anterograde amnesia or transient amnesia or memory lose” on COVID-19 vaccination. A total of 21 articles were retrieved. Of these, 10 articles were excluded after detailed screening as the did not meet the inclusion criteria. The remaining 11 articles were considered eligible. Among the eleven included articles, which were from the United Kingdom, Korea, Germany, Taiwan, Israel, Qatar, Indonesia, China and India, most studies were conducted between March 2021 and 2022.

Generally, vaccination elicits strong proinflammatory cytokine expression and the response of T cells [20,21]. The COVID-19 vaccine also demonstrated similar effects. Soon after vaccination, antigens are recognized as potential pathogens by conserved pathogens and damage-associated molecular patterns (DAMPs), whose pattern recognition receptors are found on locally or peripherally circulating immune cells (e.g., monocytes and macrophages) and stromal cells [21]. Peripheral proinflammatory cytokines (i.e., interleukin-1, interleukin-6, TNF-α, and PG-E2) expressed after vaccination are considered to be important because they may reach the brain and partly result in neuroinflammation after microglia activation. This also depends on the immunogenetic background and innate immune memory [21]. These may mimic responses to natural infections. In addition, the pathogenesis of COVD-19 vaccine-related complications has been proposed. Spike proteins translated from the mRNA COVID-19 vaccine and expressed by cells may trigger inflammatory responses similar to those induced by SARS-CoV-2 leading to these neurological complications [22,23,24]. However, elevations of inflammatory markers (e.g., cytokines) have not been detected in many cases of COVID-19 vaccine-associated encephalopathy [25] (Table 1).

It is worth noting that in the early stage of adverse reactions on COVID-19 vaccination, patients’ MRIs were normal. Cerebral fissures, cerebral cisterns and sulci were normal in appearance, without mid-line structure deviation and evidence of intracranial hemorrhage. In addition to memory loss, more attention should be paid to autoimmune encephalitis cases, which were characterized by onset of symptoms of encephalitis within 7 to 30 days, followed by recurrent seizures and progressive cognitive decline over a 4-week period after COVID-19 vaccination. Lymphocytic pleocytosis, elevated protein and glucose levels were also found on CSF examination [9,10,13]. As a post-vaccination phenomenon, unexplained autoimmune encephalitis has been described after COVID-19 vaccination. Neuroimaging results were unremarkable, but pleocytosis was found in the CSF of all patients. However, all patients responded well to corticosteroids [10,26]. Autoimmune encephalitis is an infrequent, newly described group of neurological inflammation diseases of the central nervous system commonly associated with specific autoantibodies. Typical autoimmune encephalitis includes subacute deficits of memory and cognition—impaired memory over a period of days to weeks. Although acute disseminated encephalomyelitis (ADEM) has been reported following inactivated COVID-19 vaccination, such cases were different to typical ADEM in numerous ways [27]. The initial MRI lesions were limited to the medial temporal lobe (MTL) and insular cortical, where white matter or basal ganglia with the typical MRI feature of limbic encephalitis was excluded. On the other hand, most ADEM cases show bilateral confluent white matter lesions in both cerebral hemispheres in the early stage of virus infection [28].

Although the adverse effect of transient amnesia is uncommon after COVID-19 vaccination, these reports all showed unexpected onset of autoimmune encephalitis. These cases describe hyperactive acute encephalopathy following vaccination. Antiphospholipid syndrome may be associated with viral infections or vaccinations, including COVID-19 vaccine [29,30,31,32]. However, the relationship between antiphospholipid syndrome COVID-19 vaccination is still ambiguous [33]. The key mechanism may be cross-reactivity among antigenic epitopes and self-epitopes present in adenoviral vectors or receptors of mRNA-based COVID-19 vaccine formulations [34]. Therefore, we may link the development of acute encephalopathy to the COVID-19 vaccine because patients showed a transient relationship between the two and had no other risk factors of encephalopathy. Thus, clinicians should consider autoimmune encephalitis to differentiate such diagnosis when assessing post-vaccine neurologic symptoms. Additionally, autoimmune encephalitis is treatable by corticosteroid, levetiracetam, oxcarbazepine, methylprednisolone, rituximab, Cavit-D3, mycophenolate mofetil, dexamethasone or immunosuppressive therapy with steroids. For autoimmune dementia, oral memantine, donepezil and clopidogrel may be helpful for the treatment of memory loss. The symptoms of those patients were gradually improved after treatment with oral administration in two weeks and no brain damage and severe sequela were involved. Fortunately, most patients responded well to therapy and exhibited a benign course of the disease, with almost complete recovery from neurological symptoms. Thus, further studies will be needed to confirm this conclusion.

Neurological adverse effects have been reported in various SARS-CoV-2 vaccinations. Previous studies regarded the neurological disorder of memory loss and clinical diagnostics in patients after COVID-19 vaccination. Most patients presented with amnesia and other disorders that were diagnosed post-vaccinal encephalitis by imaging modalities or laboratory surveillance. In such cases, there was no definitive evidence to support the relationship between the COVID-19 vaccine and short-term memory loss with autoimmune encephalitis. Despite this, there are recognized rare adverse events that have been causally linked to SARS-CoV-2 vaccines; it might be useful to point out more than with the currently available data to address the relationship between the COVID-19 vaccine and short-term memory loss with autoimmune encephalitis. However, the current advice is that the benefits of the vaccination outweigh the risk. This appears to be accurate from a neurological standpoint. Currently, population worldwide are receiving COVID-19 booster doses. We believe that this may resulting in an increasing number of cases and more articles to determine the mechanism of AE caused by COVID-19 vaccination. Although our study could not provide and establish a possible causality of encephalitis by COVID-19 vaccination, further study is needed in the future.

## 4. Conclusions

Since billions of people worldwide have been vaccinated, some cases of neurological conditions may occur post-vaccination simply by chance. Such cases included in our report should increase awareness of possible rare autoimmune reactions following this novel vaccination. In summary, short-term memory loss with autoimmune encephalitis is rare and its causes are still mostly unknown and are only now being determined. Autoimmune encephalitis is an under-recognized condition and has a favorable prognosis if treated promptly. Furthermore, we still believe that the benefits of COVID-19 vaccination outweigh the risks of ongoing vaccination programs.

## Figures and Tables

**Table 1:** List of reviewed studies regarding the neurological disorder of memory and clinical diagnostics in patients after COVID-19 vaccination.

Country	Case	Vaccine	Neurological Disorder of Memory	Clinical Diagnostics	Post-Vaccinal Encephalitis	Ref.
**United Kingdom**	38-year-old female	First dose of the Pfizer-BioNTech SARS-CoV-2 vaccine	Ongoing difficulties with short-term memory.	The blood pressure, glucose level, and heart rate were found to be normal, as were electrocardiogram and brain CT data.	Functional neurological disorder.	[8]
	36-year-old female	First dose of Moderna SARS-CoV-2 vaccine	Functional weakness, tremor likely due to anxiety, and fatigue symptoms similar to those associated with chronic fatigue syndrome.	MRI of the brain and spine were normal, as were electromyography and nerve conduction velocity studies.	Chronic fatigue syndrome.	[8]
**Korea**	57-year-old female	Two doses of the AstraZeneca ChAdOx1-S vaccine	Cognitive decline including attention and memory deficits along with gradually worsening dysphasia.	A cerebrospinal fluid (CSF) study was normal. However, magnetic resonance imaging (MRI) of the brain demonstrated restricted diffusion along the left insular and mesial temporal cortices with corresponding hyperintensity on fluid-attenuated inversion recovery (FLAIR) without contrast enhancement.	The follow-up CSF study revealed pleocytosis (22/μL, lymphocytes 91%), glucose of 114 mg/dl, elevated protein as 88.3 mg/dl, a positive oligoclonal IgG band. Autoimmune encephalitis.	[9]
**Germany**	21-year-old female	First dose of the AstraZeneca ChAdOx1-S vaccine	Subacute onset of working memory impairment, impaired mental status, or psychiatric symptoms.	Emergency brain magnetic resonance imaging (MRI) was performed with normal status of the parenchyma. The extensive diagnostic workup remained negative, including chest X-ray, sonography of the abdomen, and serological examinations of serum and cerebrospinal fluid (CSF).	A lumbar puncture on the day of admission revealed lymphocytic pleocytosis of 46 leukocytes/μL. Autoimmune encephalitis.	[10]
	63-year-old female	First dose of the AstraZeneca ChAdOx1-S vaccine	Subacute onset of working memory impairment, impaired mental status, or psychiatric symptoms.	MRI showed normal status of the parenchyma.	An encephalitis was diagnosed due to the result of lumbar puncture showing a lymphocytic pleocytosis of 115 leukocytes/μL. Autoimmune encephalitis.	[10]
	63-year-old male	AstraZeneca ChAdOx1-S vaccine	Presented with isolated aphasia.	MRI of the brain was normal; in particular, no evidence of ischemia or herpes encephalitis.	A lumbar puncture was performed showing a pleocytosis of 7 leukocytes/μL. Autoimmune encephalitis.	[10]
**Taiwan**	Age from 42 to 74, 4 females and 6 males	Moderna COVID-19 (mRNA-1273) vaccine	Suspected stroke, transient amnesia, facial nerve palsy, weak hands and feet.	N/A	N/A	[11]
**Israel**	48-year-old male	Second dose of Pfizer-BioNTech SARS-CoV-2 vaccine	Memory deficits and anterograde amnesia. Severe impairments in short-term memory, temporal orientation, abstraction and language skills.	Electroencephalogram and neurological exam were normal apart from a Montreal Cognitive Assessment (MoCA) score. Cranial magnetic resonance imaging (MRI) showed hyper intense signal on both medial temporal lobes (more on the left) including the parahippocampal gyrus on T2-weighted fluid-attenuated inversion recovery and diffusion-weighted imaging.	CSF cultures were negative and demonstrated normal protein and glucose without pleocytosis.	[12]
**Qatar**	32-year-old male	Moderna COVID-19 (mRNA-1273) vaccine	Disoriented and amnesic, and onset of forgetfulness and mood disturbance within 24 h of receiving the COVID-19 vaccine dose without remembering what happened after that.	MRI of the brain did not reveal any acute or chronic abnormality.	Lumbar puncture with cerebrospinal fluid (CSF) study was performed, and it showed elevated protein levels (0.76 gm/L, reference range = 0.15–0.45) with average cell counts (white blood cells of 3 u/L) and glucose levels. Acute encephalopathy.	[13]
**Indonesia**	44-year-old male	Sinovac vaccine	After vaccination, the patient had difficulty communicating verbally. The complaints of difficulty concentrating, and forgetting were getting worse, accompanied by incoherent speech. The patient also had cognitive deficits. The patient’s neurobehavioral status showed short-term memory impairment and impaired concentration.	Brain MRI showed chronic cortical infarct in left temporal lobe and multiple subacute lacunar infarcts in the left corona radiata, left basal ganglia and left frontal lobe. The ENMG results later showed motor and sensory polyradiculoneuropathy on the upper and lower extremities, leading to the diagnosis of chronic inflammatory demyelinating polyradiculoneuropathy (CIDP).	Secondary antiphospholipid syndrome and autoimmune dementia.	[14]
**Taiwan**	82-year-old female	First dose of the mRNA-1273 SARS-CoV-2 vaccine (Moderna)	Memory impairment, loss of attention and concentration, murmuring, and unsteadiness.	Brain MRI revealed hyperintense signal on fluid-attenuated inversion recovery (FLAIR) sequence imaging and abnormal gyral enhancement on T1-weighted imaging in the right middle and posterior temporal lobe with no evidence of myelitis.	CSF analysis showed no pleocytosis but elevated CSF protein. CSF rapid plasma reagin (RPR), treponema pallidum hemagglutination (TPPA) immunoelectrophoresis, and cytology were negative.	[15]
**Taiwan**	55-year-old male	First dose of the AstraZeneca ChAdOx1-S vaccine	Impaired verbal expression, progressive disorientation to people and place and slow response.	Magnetic resonance angiography of brain was showed pachymeningeal enhancement without definite abnormal signal intensity over brain parenchyma.	CSF testing showed the white cell count was 16/μL, with a neutrophil/lymphocyte/monocyte count 3/4/7, red cell count was 1/μL, the protein level was 97.3 mg/dL and positive antinuclear antibody. Serum white cell count, platelet count and C-reactive protein were within normal range.	[16]
**China**	24-year-old female	SARS-CoV-2 vaccine (Vero cells)	Neurological examination showed somnolence, memory decline and poor memory persisted.	Initial brain MRI showed abnormal signals in the bilateral temporal cortex.	The WBC count of CSF was 51 × 10^6^/L. CSF was negative for antibodies to major pathogens and cultures of bacteria and fungi; high-throughput genome sequencing also revealed no pathogens.	[17]
**India**	65-year-old male	First dose of the AstraZeneca ChAdOx1-S vaccine	Cognitive deficits, memory impairments and sudden memory loss. Unable to describe both short and long-term memory previously acquired.	Non-contrast CT was essentially normal with no evidence of hemorrhage or focal lesion.	All routine blood tests were normal.	[18]

## Data Availability

Not applicable.
